# Speech profile in patients with Huntington's Disease: cognitive, clinical, and sociodemographic correlations

**DOI:** 10.1590/2317-1782/e20240013en

**Published:** 2025-08-15

**Authors:** Nathália Vescia Bauer, Maria Eduarda Soares Machado, Maiara Laís Mallmann Kieling Peres, Raphael Machado de Castilhos, Maira Rozenfeld Olchik

**Affiliations:** 1 Curso de Fonoaudiologia, Universidade Federal do Rio Grande do Sul – UFRGS - Porto Alegre (RS), Brasil.; 2 Programa de Pós-Graduação em Medicina: Ciências Médicas, Universidade Federal do Rio Grande do Sul – UFRGS - Porto Alegre (RS), Brasil.; 3 Serviço de Neurologia,Hospital de Clínicas de Porto Alegre – HCPA - Porto Alegre (RS), Brasil.; 4 Departamento de Cirurgia e Ortopedia, Faculdade de Odontologia, Universidade Federal do Rio Grande do Sul – UFRGS - Porto Alegre (RS), Brasil.

**Keywords:** Huntington's Disease, Speech, Dysarthria, Cognition, Acoustic Analysis

## Abstract

**Purpose:**

To describe speech profiles in individuals with Huntington's Disease (HD), correlate them with cognitive and clinical aspects, and compare them with healthy controls.

**Methods:**

Symptomatic individuals with a clinical and molecular diagnosis of HD were included. Seven healthy controls, matched by age and sex, were also included. Clinical and sociodemographic data were obtained from medical records. The Unified Huntington's Disease Rating Scale was used to measure severity. Cognitive data were collected using verbal fluency, symbol digit modalities, and Stroop tests. Auditory perceptual assessments were used to evaluate speech, and acoustic analysis extracted information about the following tasks: sustained vowel /a/, utterances with different intonations, oral diadochokinesis, spontaneous speech, and the repeated diphthong /ju:/.

**Results:**

Of the seven individuals with HD, four women with a mean age of 48.86 (±16.03), presented severe (57.15%), moderate (28.57%), and mild (14.28%) dysarthria. Speech impairment in HD case subjects was related to overall motor decline; the worse the motor symptoms, the worse the speech impairment. There was no correlation with the other clinical data or cognition. The case subjects were significantly worse than the control group, specifically regarding the subsystems of phonation (fundamental frequency, phonation time, local jitter, local shimmer), respiration (maximum phonation time) and articulation (speech rate, phonation time in spontaneous speech, number of syllables in spontaneous speech, average duration of syllables and duration of spontaneous speech).

**Conclusion:**

In HD subjects, the most affected speech subsystems were articulation, phonation, and respiration. Poor motor speech patterns were associated with overall motor decline. Speech assessments may provide biomarkers that predict HD progression.

## INTRODUCTION

Huntington's disease (HD) is an autosomal dominant neurodegenerative condition characterized by neuropsychiatric and behavioral symptoms^(1)^. In Brazil, the national prevalence of HD is unknown. However, in the state of Rio Grande do Sul, research has revealed a minimum prevalence of 1.85/100,000^(2)^, which is lower than in European countries but similar to other Latin American countries^(3,4)^.

This disorder is caused by CAG (cytosine-adenine-guanine) repeat expansions in exon 1 of the HTT gene, located on the short arm of chromosome 4 (4p16.3)^(5)^. In normal individuals, CAG repeats range from 10 to 35; however, HD patients may have an allele with 36 to 60 CAG repeats. Since HD is inherited in an autosomal dominant pattern, one allele with CAG repeat expansions is sufficient to cause the disease^(6)^. The age of onset varies and depends largely on the number of CAG repeats^(7)^. Affected individuals become symptomatic, on average, at 35 - 44 years of age^(8)^.

As the disease progresses, cognitive impairment, such as reduced planning ability, becomes more pronounced. Initially, memory is one of the least affected functions, but eventually, subcortical dementia syndrome sets in. More recently, imaging tests have shown that subcortical involvement can cause cortical deficits^(9)^. Depression and anxiety are common, and the suicide rate is high among individuals with HD^(10)^. One study estimated that more than 25% of individuals living with HD attempt suicide at some point during the course of the condition^(11)^.

HD-related speech disorders are common, with dysarthria estimated at a 93% to 100% prevalence. Changes in the cortico-basal ganglia-thalamo-cortical loop cause involuntary movements and speech symptoms that are broadly classified as hyperkinetic dysarthria^(12-17)^. This subtype is characterized by prolonged intervals, varying or reduced articulation speed, imprecise consonants, and frequent changes in intensity^(18-21)^. It occurs in 20% of adults with a diagnosis of dysarthria^(22)^. Hertrich and Ackermann reported increased acoustic variability and voice-onset time, in addition to the excessive prolongation of short vowels^(23)^. Skodda et al. identified a pattern of reduced articulation rate, increased pauses, and difficulty in producing single syllables^(24)^. Rusz et al. detected irregular fluctuations in tone, sudden interruptions in phonation, and poor articulation. The authors noted a moderate correlation (r = -0.48) between sudden interruptions in phonation and voluntary domains of the Unified Huntington's Disease Rating Scale (UHDRS)^(25)^.

In clinical settings, auditory-perceptual assessment (APA) is considered the gold standard for evaluating speech and speech disorders^(26)^. While there may not be a specific protocol for every clinical assessment for dysarthria, patients are typically asked to repeat words and phrases and perform other speech tasks^(27,28)^. As a complement to APA, acoustic assessments have proven to be non-expensive, non-invasive, and easy to perform, with the aid of software^(22)^. Data from these tests can serve as diagnostic support, and qualify interpatient and intrapatient comparisons^(29,30)^. Furthermore, computerized acoustic assessments can provide objective information that the human ear cannot detect, increasing the contribution to studies regarding speech biomarkers in neurodegenerative diseases^(31,32)^.

The current literature on speech in symptomatic HD patients has been limited, particularly regarding the acoustic variables of these patients' speech subsystems or speech profiles, within the Brazilian population. By using controls matched by sex and age, this study aims to describe the speech characteristics of patients with HD and correlate them with clinical, cognitive, and sociodemographic aspects.

## METHODS

This was a cross-sectional study.

### Participants

The participant group was a convenience sample of symptomatic patients from the neurogenetics outpatient clinic at Hospital de Clínicas de Porto Alegre (HCPA), Rio Grande do Sul. Subjects with a clinical and molecular diagnosis of HD were included. Healthy controls were matched by age and sex to case subjects. Subjects were excluded from either group if they were younger than 18, had a history of other neurological events, sensory disorders, or other systemic diseases or structural changes that affect speech or voice. The ethics committee approved and identified the project under number 2019-0648. All subjects gave their informed consent by signing a form.

### Clinical and sociodemographic data

Clinical and sociodemographic data were collected from electronic medical records on the same day as the speech-language assessment. The variables were age, sex, disease history, age of onset, time since diagnosis, current neurological status, education level, and the number of CAG repeat expansions.

### Clinical assessment

The Unified Huntington's Disease Rating Scale (UHDRS)^(33)^ is the most widely used instrument to monitor the progress of patients with HD^(34,35)^. It consists of 83 items divided into four domains:

- Motor assessment: 31 items address various aspects of motor function. Each item has five options, from 0 to 4. A score of 4 indicates greater motor impairment.
- Cognitive assessment: three tests evaluate cognitive capacity - verbal fluency, the Symbol Digit Modalities Test, and Stroop tests. The higher the sum of the correct answers, the better the performance.
- Behavioral assessment: neuropsychiatric HD symptoms are given severity and frequency scores (from 0 to 4, with 4 being the most severe), to calculate the sum.
  d) Functional assessment: three scales are completed (1) List of daily life tasks – 1 point is given for each activity the patient is still able to do. The higher the sum, the higher the capacity; (2) Independence - scores range from 10 (bedbound) to 100 (no need for special care); (3) Total functional capacity - five functional aspects (employment, finances, domestic chores, activities of daily living and care level) are examined. Scores range from 0 to 13, with 13 being normal.

### Cognitive assessment

The Frontal Assessment Battery (FAB) evaluates cognitive functions such as phonemic fluency, cognitive flexibility, inhibitory control, and sensitivity to interference. The maximum score for each subtest is three points. Higher scores indicate better performance, and the total test score is calculated by adding the scores of the six subtests (maximum score = 18). It is validated in Brazilian Portuguese^(36)^.

The Stroop Color and Word Test (SCWT) assesses inhibitory control and attention. There is a reading task, a color naming task, and an interference task during which the individual must read the color in which the word is written, even though there is a mismatch between the ink and the words. The scores of the items completed in 120 seconds are calculated. The higher the score, the better the performance^(37)^.

The Montreal Cognitive Assessment (MoCA) is a cognitive screening test that examines visuospatial apraxia, naming, memory, attention, language, abstraction, and orientation. A score of 26 or higher suggests preserved cognition^(38)^.

The Symbol Digit Modalities Test (SDMT) is commonly used to assess psychomotor speed (processing and motor speed). Attention, visual scanning and tracking, and working memory affect scores^(39)^.

### Speech assessment

#### Data collection

The speech tasks were recorded in a single session, using Audacity software, an Andrea Pure audio USB adapter, and a KARSECT HT-9 microphone positioned approximately 5cm from the patient's mouth. Case subjects and controls recorded 16-bit speech samples at a 44.1 kHz rate in a silent environment with no soundproofing. Both groups were asked to perform five tasks: (a) sustain the vowel /a/ in a single breath, for as long as possible, (b) repeat the diphthong /ju:/ in a single breath, (c) say /pataka/ as quickly as possible in a single breath (DDK) (d) use the correct intonation to say the sentence “It rained a lot this weekend” as a statement, a question and an exclamation, (e) spontaneously answer the question “What have you done today since waking up?” for 60 seconds.

#### Auditory perceptual assessment (APA)

This is currently the gold standard for assessing dysarthria. Three trained speech-language pathologists with at least five years of experience rated the blinded voice samples, using a kappa value of ≥ 0.90 for interrater agreement. A simulation activity preceded the assessment for the purpose of training. The examiners listened to the blinded speech samples in random order and rated the speech subsystems (phonation, articulation, respiration, resonance, and prosody). Using Duffy’s classification, each sample was rated as (0) normal, (1) mildly impaired, (2) moderately impaired, or (3) severely impaired. Subsequently, the final diagnosis was expressed as (0) normal, (1) mild dysarthria, (2) moderate dysarthria, or (3) severe dysarthria.

#### Acoustic analysis

**Figure 1:**
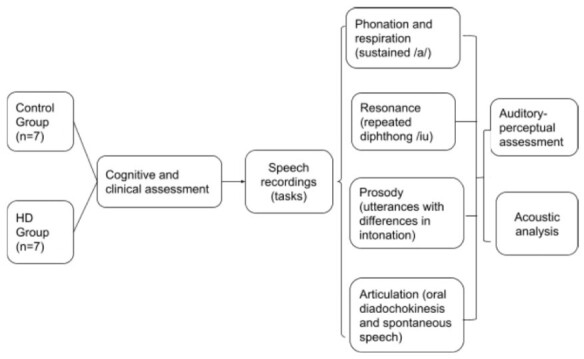
Experimental design

Acoustic analysis was performed in Praat^(40)^ (version 6.1.55) software, with a script^(41)^ to automatically detect intensity peaks. Syllable structure in Brazilian Portuguese only allows vowels at the nucleus; thus, counting intensity peaks is the same as determining the number of syllables. De Jong and Wempe^(41)^ were used to check reliability by comparing the results of manual analyses with those of the Praat script. The acoustic parameters recommended by Rusz et al.^(42)^ and Vogel and Maruff^(43)^ were also used. For phonation, we extracted information regarding jitter (rap), shimmer (local), fundamental frequency (F0 in Hz), standard deviation of the fundamental frequency (F0 SD) and the harmonics-to-noise ratio (HNR), measured through the sustained vowel /a/. For articulation, DDK and spontaneous speech recordings were used to analyze the number of syllables, number of pauses, total duration (in seconds), phonation time (total duration minus pauses), phonation rate (phonation time divided by total duration), speech rate (number of syllables divided by total duration), articulation rate (number of syllables divided by phonation time), average syllable duration (ASD) and number of pauses weighted by total time. From the repeated [ju:] task, the ratio between the second formant for the vowel [i] and the 2nd formant for the vowel [u] was used as a measure of vowel centralization. This measure can indicate reduced articulatory amplitude. Although it is not an exclusive respiratory measure, the maximum phonation time (MPT) was used to assess the respiratory subsystem, due to the association with the myoelastic-aerodynamic model of phonation. Regarding prosody, variations in F0 and intensity were evaluated during statement, question, and exclamation utterances. Variations in the F0 - the difference between the maximum and minimum values ​​of the F0 - indicate melodic changes and, therefore, the speaker's ability to vary intonation (Figure 1).

### Statistical analysis

A qualitative analysis of the data was performed using interquartile ranges and medians. Quantitative data were analyzed using Spearman's rank correlation coefficient. To compare groups, means and standard deviations were calculated, and the Student’s t-test was applied.

## RESULTS

The seven case subjects included in the study - four females and three males - were matched by age and sex with healthy controls. Table 1 describes the clinical and sociodemographic characteristics of the sample. No statistically significant differences were found between the groups. Table 2 presents the results of the clinical severity scales for functional, behavioral, independence (Unified Huntington's Disease Rating Scale), and cognitive capacities. Table 3 describes the auditory-perceptual speech assessment. All case subjects presented impaired prosodic modulation and imprecise articulation; six presented speech and breathing discoordination, and slow speech rates. Table 4 shows the acoustic analysis of speech subsystems in the cases and controls (phonation, respiration, and prosody). The case subjects performed significantly worse than the control group, specifically regarding the variables of fundamental frequency, maximum phonation time, jitter (local), shimmer (local), variations in fundamental frequency in statements, and variations in intensity in questions and exclamatory utterances. Table 5 shows the acoustic analysis of speech tasks in the cases and controls. The HD subjects presented significantly worse results than the control group, specifically regarding the variables of speech rate, phonation time in spontaneous speech, number of syllables, average duration of syllables, and duration of spontaneous speech. In Table 6, we show correlations of significance between speech and clinical aspects.

**Table 1:** Clinical and demographic variables

Variable	Q1	Median	Q3
Age	40	48	64
Education	7	9	11
CAGexp	43	47	47
Age of onset	30	45	50
Disease duration	3	6	9

**Caption:** Q1 = first quartile; Q3 = third quartile; CAGexp = CAG repeat expansions

**Table 2:** Clinical severity scales

Variable	Q1	Median	Q3
UHDRS motor assessment	30	41	68
UHDRS behavioral assessment	4	11	37
Total functional capacity	8	8	18
Independence scale	60	70	70
Functional assessment	3	4	5
Phonological fluency	9.5	11	13
Semantic fluency	6	8	9.5
MoCA	10	12.5	18.5
Symbol digit modalities	0.75	8.5	24.25
FAB	4.75	8.5	10.5
Stroop word reading	26	38.5	90.75
Stroop color naming	27.5	46	88
Stroop interference trial	11	12.5	32.5

**Caption:** Q1 = first quartile; Q3 = third quartile; UHDRS = Unified Huntington’s Disease Rating Scale; MoCA = Montreal Cognitive Assessment; FAB = Frontal Assessment Battery

**Table 3:** Results of the auditory-perceptual assessment

**1**	**Sex**	**Age(years)**	**Education (years)**	**Disease duration (years)**	**Age of onset (years)**	**Dysarthria severity**	**Speech characteristics**	**% Intelligibility**
**HD 1**	**Male**	**64**	**9**	**14**	**50**	**Severe**	monopitch, monoloudness, impaired prosodic modulation, hypophonia, speech and breathing discoordination, imprecise articulation, slow speech rate	**50%**
**HD2**	**Female**	**40**	**11**	**10**	**30**	**Moderate**	impaired prosodic modulation, speech and breathing discoordination, imprecise articulation, slow speech rate	**60%**
**HD3**	**Female**	**48**	**11**	**3**	**45**	**Mild**	impaired prosodic modulation, imprecise articulation	**75%**
**HD4**	**Female**	**54**	**8**	**4**	**50**	**Severe**	monopitch, monoloudness, impaired prosodic modulation, hypophonia, speech and breathing discoordination, imprecise articulation, slow speech rate, prolonged phonemes, dysfluency	**50%**
**HD5**	**Male**	**23**	**7**	**7**	**16**	**Severe**	monopitch, monoloudness, impaired prosodic modulation, hypophonia, speech and breathing discoordination, imprecise articulation, slow speech rate	**45%**
**HD6**	**Male**	**42**	**11**	**6**	**36**	**Moderate**	abnormal voice, impaired prosodic modulation, hypophonia, speech and breathing discoordination, imprecise articulation, slow speech rate	**50%**
**HD7**	**Female**	**71**	**5**	**5**	**66**	**Severe**	impaired prosodic modulation, speech and breathing discoordination, imprecise articulation, slow speech rate, prolonged phonemes	**45%**

**Table 4:** Acoustic analysis of phonation, respiration and prosody in HD case subjects and healthy controls

**Speech subsystem**	**Variables**	**Cases**	**Controls**	**p-value**
Phonation	Jitter (local)	0.87(±0.49)	0.39(±0.10)	0.04
	Shimmer (local)	8.47(±4.92)	3.59(±2.72)	0.046
	Average FF	215.99(±70.15)	154.53(±43.21)	NS
	Minimum FF	174.45(±63.13)	149.62(±43.04)	NS
	Maximum FF	297.78(±108.60)	160.42(±44.13)	0.015
	Standard deviation of FF	21.88(±21.24)	1.62(±0.50)	0.045
	HNR	17.32(±7.16)	20.75(±4.23)	NS
Respiration	MPT	5.45(±5.03)	17.22(±4.43)	0.001
Prosody	Changes in F0 - statements	221.26(±112.95)	461.62(±176.52)	0.012
	Changes in intensity - statements	48.58(±20.37)	30.43(±6.52)	NS
	Changes in F0 – questions	194.36(±111.61)	361.67(±197.63)	NS
	Changes in intensity - questions	47.68(±18.73)	29.39(±5.92)	0.042
	Changes in F0 - exclamations	206.93(±132.22)	390.41(±184.24)	NS
	Changes in intensity - exclamations	47.72(±13.78)	33.03(±9.65)	0.042

**Caption:** F2 = second formant frequency; F2i/F2u = second formant frequency for the vowel [i] divided by the second formant frequency for the vowel [u] in seconds; FF= fundamental frequency; MPT = maximum phonation time; HNR = harmonics-to-noise ratio; ASD = average syllable duration in spontaneous speech; NS = not statistically significant

**Table 5:** Acoustic analysis of speech tasks in HD case subjects and healthy controls

	Variables	Cases	Controls	p-value
Diphthong, repeated /ju:/	F2I/F2U	2.37(±0.48)	2.52(±0.30)	NS
Oral diadochokinesis /pataka/	Number of syllables	22.86(±24.15)	48.57(±13.60)	0.035
	Number of pauses	2.00(±2.08)	0.29(±0.76)	NS
	Duration	6.38(±4.63)	9.65(±2.72)	NS
	Phonation time	5.05(±4.63)	9.55(±2.58)	0.05
	Speech rate	3.39(±1.15)	5.28(±1.60)	0.028
	Articulation rate	4.34(±0.53)	5.30(±1.56)	NS
	ASD	0.23(±0.03)	0.21(±0.07)	NS
Spontaneous speech	Number of syllables	76.86(±25.58)	195.29(±36.82)	<0.0001
	Number of pauses	10.43(±2.23)	12.71(±6.60)	NS
	Duration	30.86(±1.77)	59.78(±0.59)	<0.0001
	Phonation time	17.72(±6.11)	49.98(±5.86)	<0.0001
	Speech rate	2.49(±0.83)	3.27(±0.62)	NS
	Articulation rate	4.44(±0.88)	3.89(±0.48)	NS
	ASD	0.23(±0.05)	0.26(±0.03)	NS

**Caption:** ASD = average syllable duration in spontaneous speech; NS = not statistically significant

**Table 6:** Acoustic values of Spearman’s rank correlation coefficient

Variables	Education (years)	CAGexp	Duration (years)	UHDRS motor function
	Spearman’s rank correlation (ρ)			
APA of dysarthria severity	-0.868 (0.011)	NS	NS	NS
F2[i]	NS	NS	NS	-0.821 (0.023)
F0MAX	NS	NS	NS	-0.929 (0.003)
HNR	NS	0.852 (0.015)	NS	NS
Oral diadochokinesis				
Number of syllables	NS	NS	NS	-0.800 (0.031)
Speech rate	NS	NS	NS	-0.857 (0.014)
Spontaneous speech				
Number of pauses	NS	NS	NS	NS
Minimum intensity - question	0.778 (0.039)	NS	NS	NS
Minimum intensity - exclamation	NS	NS	NS	-0.893 (0.007)
Changes in intensity - exclamation	NS	NS	0.775 (0.041)	0.929 (0.003)
MPT	NS	NS	NS	-0.893 (0.007)

**Caption:** APA = auditory-perceptual assessment; F2[i] = second formant frequency for the vowel [i]; F0MAX = maximum fundamental frequency; HNR = harmonics-to-noise ratio; MPT = maximum phonation time; NS = not statistically significant

## DISCUSSION

In this study, all HD case subjects were diagnosed with some degree of dysarthria, with 4 (57.15%) having severe speech impairment, 2 (28.57%) moderate impairment and 1 (14.28%) mild impairment. The most frequent changes detected in the auditory perceptual assessment were impaired prosodic modulation, imprecise articulation, speech and breathing discoordination, and slow speech rate.

The case group presented significantly worse acoustic parameters than the control group regarding phonation (fundamental frequency, phonation time, jitter (local), shimmer (local), respiration (maximum phonation time) and articulation (speech rate, phonation time in spontaneous speech, number of syllables in spontaneous speech, average duration of syllables and duration of spontaneous speech).

The literature^(21,23,24,28)^ that addresses affected speech in HD patients has described language, used discourse analysis, and evaluated speech subsystems. All the articles analyzed the speech of individuals in early stages of the disease and used different software for auditory-perceptual or acoustic assessment. They describe speech profiles with increased speech onset time, vowel prolongation, slower articulation and speech rates, sudden interruptions in phonation, and imprecise articulation.

Hertrich and Ackermann^(23)^ performed an acoustic evaluation of the speech of 13 HD patients (most in more advanced stages of the disease) and 12 controls. The former group showed increased acoustic variability, speech onset time (voice onset time), and prolonged short vowels. The authors interpreted these findings as symptoms of advanced disease, which were consistent with the literature on degenerative cerebellar disorders. In our study, even though the sample size was smaller, the case subjects also presented more pauses during speech, shorter phonation times, and fewer syllables per second, corroborating Hertrich and Ackermann’s findings.

Another study evaluated 21 HD subjects (with an average disease duration of 5 years) and 21 controls. In the former group, the authors noted slower speech (slower articulation rate), increased pauses, and significant difficulty in generating single syllables^(24)^. Our research also revealed slower speech in the HD group (slower articulation rate). However, the number of pauses in articulation was significantly higher when compared to other studies. Our hypotheses for these findings include the group’s low level of education, associated with cognitive and linguistic changes. Furthermore, age, age at onset, and duration of the disease may similarly influence these variables.

There were correlations between the clinical aspects of HD and the speech profiles, such as the correspondence between the motor scale scores and the speech subsystems. In our sample, the worse the subject's overall motor assessment, the greater the impairment in all speech subsystems. There was no correlation between the speech profiles and cognitive or other clinical variables (duration of disease, age at onset, behavior, functional status, or independence).

Rusz et al.^(25)^ evaluated 34 individuals with HD (with a mean disease duration of 5.9 years) and 34 controls. They observed irregular intonation fluctuations, sudden interruptions in phonation, and imprecise articulation. They found a moderate correlation (r = -0.48) between sudden interruptions in phonation and voluntary components of the UHDRS scale. We found a significant decline in the rate of syllables per second, resulting in imprecise articulation, variations in jitter and shimmer, and compromised phonation.

Illes^(44)^ analyzed spontaneous speech in three groups: 10 subjects with Huntington's disease, 10 with Alzheimer's disease (AD), and 10 with Parkinson's disease (PD). He reported that patients with HD present verbal paraphasias in spontaneous speech and simplify complex sentences. These results corroborate our finding of significantly shorter spontaneous speech in HD patients.

As well as HD-associated language deficits, the literature has widely described reduced lexical fluency and important communication difficulties. Similarly, cognitive impairment has been studied at different stages of the disease, and changes in memory, executive function, and attention have been documented, even prior to motor symptoms^(45,46)^. The results from our study reflect these findings. Our case subjects achieved a median score of 12.5 points in the MoCA screening test^(47)^, which is below the cutoff point that distinguishes healthy adults from patients with dementia (15 points) (sensitivity 90%, specificity 77%). The FAB battery^(48)^ scores were similarly low in the HD group, with case subjects achieving a median score of 8.5 (shown in Table 2), while the cutoff point is 13.0 (±2.3). Changes in verbal fluency (both semantic and phonological) may also have impacted speech rate.

The DDK task has been cited as important in identifying neurological cases^(49)^, since affected individuals produce fewer syllables per breath^(30)^. Phonation, oral-motor function - including DDK (articulation) - and prosody are commonly impaired in HD subjects^(50)^. Since speech disorders in individuals with HD are directly related to the overall progression of motor symptoms, health professionals with a view to early intervention should refer patients for speech assessments. The worse the patient’s general motor symptoms, the greater the impairment in speech subsystems.

For all speech variables, the HD group performed worse than the controls, who were included in our research to provide a reference for comparison. This was especially necessary because not all speech variables have a cutoff point or limits of normality for the Brazilian population. In addition, since the sample included a series of cases, comparison with healthy controls aimed to ensure that differences were due to the neurodegenerative condition, and not other external variables like education.

The small sample size was a limitation; however, HD is a rare disorder, and only participants from one specialized center were recruited. Further studies with larger samples from more healthcare centers, and longitudinal monitoring of HD patients, are needed to establish the speech profiles of these individuals and contribute to new diagnostic and therapeutic approaches.

Our present research shows that individuals with HD can have significantly impaired articulation, respiration, and phonation, although articulation was the most affected speech subsystem in our results. Considering these changes, clinicians should be attentive to these three subsystems during assessments, since their scores may define plans of care in speech therapy.

## CONCLUSION

The most affected speech subsystems in the HD case subjects were articulation, phonation and respiration. Clinical assessments should include tasks that test these aspects, as they will also become a priority in therapy. Furthermore, HD speech profiles were directly related to the overall progression of motor symptoms. Evaluating the speech profiles of HD patients can support diagnosis, early rehabilitation, and a better quality of life. While the sample size in this study limits generalizations based on the findings, motor speech patterns present potential as biomarkers for predicting disease progression.
