# Supplementary material for: Speech profile in patients with Huntington's Disease: cognitive, clinical, and sociodemographic correlations
Source: Codas. 2025 Aug 15;37(4):e20240013. doi: 10.1590/2317-1782/e20240013en (PMC12419457; doi:10.1590/2317-1782/e20240013en)
Supplement: Translation

# Translation: Perfil de fala em pacientes com Doença de Huntington: correlações cognitivas, clínicas e sociodemográficas

**DOI:** 10.1590/2317-1782/e20240013pt

**Authors:** Nathália Vescia Bauer, Maria Eduarda Soares Machado, Maiara Laís Mallmann Kieling Peres, Raphael Machado de Castilhos, Maira Rozenfeld Olchik

**Affiliations:** 1 Curso de Fonoaudiologia, Universidade Federal do Rio Grande do Sul – UFRGS - Porto Alegre (RS), Brasil.; 2 Programa de Pós-Graduação em Medicina: Ciências Médicas, Universidade Federal do Rio Grande do Sul – UFRGS - Porto Alegre (RS), Brasil.; 3 Serviço de Neurologia,Hospital de Clínicas de Porto Alegre – HCPA - Porto Alegre (RS), Brasil.; 4 Departamento de Cirurgia e Ortopedia, Faculdade de Odontologia, Universidade Federal do Rio Grande do Sul – UFRGS - Porto Alegre (RS), Brasil.

**Keywords:** Doença de Huntingon, Fala, Disartria, Cognição, Análise Acústica da Fala

## Abstract

**Objetivo:**

Descrever o perfil de fala na Doença de Huntington (DH), correlacionar com aspectos cognitivos e clínicos, e comparar com controles.

**Método:**

Foram incluídos indivíduos sintomáticos, com diagnóstico clínico e molecular de DH e controles. Foram obtidos dados clínicos e sociodemográficos. A gravidade foi coletada pela Unified Huntington's Disease Rating Scale (UHDRS). A cognição foi avaliada pelos testes: fluência verbal, dígitos e stroop. A avaliação de fala foi feita por julgamento perceptivo auditivo e análise acústica.

**Resultados:**

Foram incluídos 7 indivíduos com DH, sendo 4 mulheres, com idade média de 48,86 (±16,03). Destes, 57,15% apresentaram disartria grave, 28,57% moderada e 14,28% leve. Sete controles saudáveis, pareados por sexo e idade, participaram do estudo. As alterações de fala dos indivíduos com DH estão relacionadas com a evolução dos sintomas motores, quanto piores os sintomas motores, pior o desempenho na fala. Com os demais dados clínicos, não houve correlação. Os indivíduos com DH foram significativamente piores comparados ao grupo controle nos subsistemas da fonação (frequência fundamental, tempo de fonação, jitter local, shimmer local), respiração (tempo máximo de fonação) e articulação (speech rate, tempo de fonação na fala espontânea, número de sílabas na fala espontânea, média de duração das sílabas e na duração da fala espontânea).

**Conclusão:**

Os subsistemas da fala mais afetados foram articulação, fonação e respiração. O perfil de fala está relacionado à progressão dos sintomas motores. A avaliação da fala tem a possibilidade de se configurar como um preditor da progressão da DH.

## INTRODUÇÃO

A doença de Huntington (DH) é uma doença neurodegenerativa autossômica dominante caracterizada por sintomas neurológicos, psiquiátricos e comportamentais^(1)^. No Brasil, a prevalência não é conhecida. No entanto, um estudo, realizado no estado do Rio Grande do Sul, encontrou prevalência mínima de 1,85/100.000^(2)^ inferior ao encontrado em países europeus, mas similar a outros países latino-americanos^(3,4)^.

A DH é causada pela expansão de uma sequência repetitiva do trinucleotídeo CAG (citosina-adenina-guanina) no exon 1 do gene *HTT,* localizado no braço curto do cromossomo 4 (4p16.3)^(5)^. Enquanto indivíduos normais apresentam sequências CAG entre 10 e 35 repetições, os afetados pela DH apresentam um alelo com 36 a 60 repetições CAG. Como a DH tem herança autossômica dominante, basta a presença de um alelo com expansão CAG para a doença se manifestar^(6)^. A idade de início da DH é variável e depende em grande parte do número de repetições CAG^(7)^. Os indivíduos portadores da mutação tornam-se sintomáticos em média aos 35-44 anos^(8)^.

Com a progressão da doença, as manifestações cognitivas, tais como perda da capacidade de planejamento, acentuam-se. Inicialmente a perda de memória não é a habilidade cognitiva mais comprometida, mas com a evolução, uma síndrome demencial subcortical por fim estabelece-se, porém, mais recentemente, com exames de imagem, foi possível verificar que o comprometimento subcortical podem causar déficits corticais^(9)^. Depressão e ansiedade são frequentes e a taxa de suicídio é grande entre indivíduos com HD^(10)^. Um estudo estimou que mais de 25% dos indivíduos com HD tentam o suicídio em algum momento da evolução da doença^(11)^.

Nos pacientes com DH, as alterações de fala são frequentes, tendo a disartria uma prevalência de 93 a 100%. Os distúrbios da fala decorrentes de alterações, que afetam os circuitos dos gânglios corticobasais-talamocorticais, causam movimentos involuntários e são amplamente classificados como disartria hipercinética^(12-17)^. A disartria hipercinética é caracterizada por intervalos prolongados, variável ou reduzida velocidade de articulação, consoantes imprecisas e variações excessivas de intensidade^(18-21)^. Esse tipo de disartria ocorre em 20% dos indivíduos adultos com disartria^(22)^_._ Hertrich e Ackermann, mostraram aumento da variabilidade acústica e do tempo para início da fala (*voice-onset-time*), além de prolongamento excessivo de vogais curtas^(23)^. Skodda e colaboradores encontraram um padrão de alteração na fala com redução da velocidade da fala (taxa de articulação), aumento das pausas e grande incapacidade na geração de sílabas únicas^(24)^. Já Rusz e colaboradores encontraram flutuação irregular nos tons, interrupção na fonação súbita e articulações mal realizadas. Neste estudo os autores observaram moderada correlação (*r* = - 0,48) entre interrupção súbita da fonação e componentes voluntários da escala Unified Huntington's Disease Rating Scale (UHDRS)^(25)^.

A avaliação clínica da disartria tem sido realizada através da análise de parâmetros de fala por meio do julgamento perceptivo-auditivo (JPA), padrão ouro para avaliação da fala^(26)^. Provas de repetição de palavras e frases, de análise do discurso e de produções orais pré-estabelecidas são algumas das tarefas utilizadas na prática clínica, sem que isso pressuponha a aplicação de protocolo específico^(27,28)^. Para complementar o JPA, temos a avaliação acústica da fala, de fácil aplicação, não invasiva, realizada por meio de softwares e com custo baixo^(22)^. Essas informações podem favorecer o diagnóstico, a comparação entre desempenhos de diferentes pacientes, além de possibilitar a avaliação da evolução de um mesmo paciente^(29,30)^. Ainda, os dados objetivos da avaliação acústica computadorizada tornaram-se opção ao oferecerem informações não percebidas pelo ouvido humano, o que tem aberto espaço para contribuir para estudos de biomarcadores de fala em doenças neurodegenerativas^(31,32)^.

Até agora, a literatura abordou de forma limitada estudos sobre a fala em pacientes sintomáticos, pesquisas que empreguem variáveis acústicas para todas as bases motoras da fala ou aquelas que tenham analisado o perfil de fala desses pacientes na população brasileira. Dessa forma, esse estudo se propõe a descrever as características de fala em pacientes com DH e correlacionar com aspectos clínicos, cognitivos e sociodemográficos comparando com controles pareados por sexo e idade.

## MÉTODO

Trata-se de um estudo transversal.

### Participantes

Realizou-se um processo de amostragem por conveniência. Foram convidados a participar do estudo pacientes do ambulatório de neurogenética do Hospital de Clínicas de Porto Alegre (HCPA),Porto Alegre - Rio Grande do Sul. Foram incluídos indivíduos sintomáticos, com diagnóstico clínico e molecular de DH que concordaram em participar do estudo. No grupo controle foram incluídos indivíduos saudáveis da comunidade pareados por idade e sexo com o grupo caso. Foram excluídos, em ambos os grupos, indivíduos menores de 18 anos ou que tivessem história de outros eventos neurológicos prévios, distúrbio sensorial que impossibilita a realização dos testes, doenças sistêmicas e/ou alterações estruturais que afetassem a voz e/ou fala. Projeto aprovado pelo comitê de ética sob número 2019-0648. Todos os participantes assinaram o Termo de Consentimento Livre e Esclarecido.

### Dados clínicos e sociodemográficos

Os dados clínicos e sociodemográficos foram coletados do prontuário eletrônico do paciente no registro da consulta médica realizada no mesmo dia da avaliação fonoaudiológica. As variáveis coletadas foram idade, sexo, histórico da doença, idade de início dos sintomas, tempo de diagnóstico, estado neurológico atual do paciente, escolaridade e o número de repetições do alelo CAG expandido.

### Avaliação clínica

O protocolo de avaliação clínica dos pacientes foi realizado do Unified Huntington's Disease Rating Scale (UHDRS)^(33)^, que é o instrumento de medida mais utilizado para acompanhar a evolução dos pacientes com DH^(34,35)^_._ É composta de 83 itens divididos em quatro partes:

- Escore motor: É calculado a partir do somatório de 31 itens relacionados a diversos aspectos da função motora. Em cada item, há cinco opções, de 0 a 4, sendo que 4 indica maior prejuízo motor.
- Escore cognitivo: A capacidade cognitiva é avaliada através de três testes: teste de fluência verbal, Symbol Digit Modalities Test e teste de Stroop. A somatória das respostas corretas nos três testes é calculada. Escores mais altos correspondem à melhor desempenho.
- Escore comportamental: A avaliação dos sintomas comportamentais é realizada através do somatório da gravidade e frequência (de 0 a 4, sendo 4 mais grave) em cada item de um questionário composto de várias manifestações neuropsiquiátricas presentes na DH.
- Escore funcional: esse escore é divido em três escalas: 1) Lista de tarefas da vida diária: Para cada item, dá-se 1 ponto quando o paciente ainda é capaz de realizá-lo. No somatório, um escore maior indica menor incapacidade; 2) Escala de independência: varia de 10 (restrito ao leito) a 100 (sem necessidade de cuidados especiais); 3) Escala de capacidade funcional total: somatório de cinco aspectos funcionais (emprego, finanças, cuidados com a casa, atividades da vida diária e nível de cuidado). Essa escala varia de 0 a 13, sendo 13 o normal.

### Avaliação cognitiva

Bateria de Avaliação Frontal (FAB): composta por tarefas que avaliam questões cognitivas tais como fluência fonêmica, flexibilidade cognitiva, controle inibitório, sensibilidade a interferências. A pontuação máxima de cada subteste é de três pontos (com pontuações mais altas indicando melhor desempenho) e a pontuação total do teste é calculada somando as pontuações dos seis subtestes (pontuação máxima=18). Está validada para o português brasileiro^(36)^.

Teste de STROOP (SCWT): tem como objetivo avaliar o controle inibitório e processos de atenção. O teste é composto por uma tarefa de leitura e outra de nomeação de cor e, por fim, pela tarefa de interferência em que o indivíduo precisa ler a cor que a palavra está escrita (cores incongruentes com o escrito). O resultado se dá a partir das três pontuações diretas com base no número de itens preenchidos em cada uma das três folhas de estímulo em 120s. Quanto maior a pontuação, melhor o desempenho do indivíduo^(37)^.

*Montreal Cognitive Assessment* (MoCA): trata-se de um teste de rastreio cognitivo, que avalia funções como apraxia visuoespacial, nomeação, memória, atenção, linguagem, abstração e orientação. Uma pontuação de 26 ou superior é esperado para indivíduos com cognição preservada^(38)^.

Teste de símbolos e dígitos (dígitos span): comumente usado para avaliar a velocidade psicomotora, mede a velocidade de processamento, bem como a velocidade motora. O desempenho também é afetado pela atenção, varredura visual e rastreamento e memória de trabalho^(39)^.

### Avaliação da fala

#### Coleta de dados

As tarefas foram gravadas por meio do software Audacity, em ambiente com isolamento acústico, utilizando o microfone KARSECT HT-9 com o adaptador Andrea Pure audio USB posicionado a aproximadamente 5 cm da boca do paciente, em sessão única, em ambiente silencioso e sem isolamento acústico, tanto para os pacientes quanto para o grupo controle. As gravações foram amostradas em 44,1 kHz e quantificadas em 16 bits. Ambos os grupos foram solicitados a realizar cinco tarefas seguindo modelo fornecido pelo pesquisador: (a) sustentação da vogal [a], em uma respiração, pelo maior tempo possível; (b) ditongo /iu/ repetido em uma única respiração; (c) diadococinesia /pataka/ o mais rápido possível em uma única respiração; (d) falar a frase “choveu muito neste fim de semana” em entonação afirmativa, interrogativa e exclamativa; (e) fala espontânea respondendo à pergunta “o que você fez hoje desde que acordou?” por 60 segundos.

#### Julgamento perceptivo auditivo

Este é atualmente o padrão ouro na avaliação da disartria. Três fonoaudiólogos treinados, com pelo menos cinco anos de experiência e cegos para o diagnóstico realizaram o JPA com índice Kappa de concordância ≥ 0,90. Antes dos procedimentos de análise de fala, foram apresentadas e avaliadas diferentes alterações de fala para o treinamento auditivo. Os examinadores ouviram as amostras de fala em ordem aleatória e analisaram os subsistemas da fala (fonação, articulação, respiração, ressonância e prosódia) com base nas dimensões descritas por Duffy, classificando cada um deles como (0) normal, (1) levemente alterado, (2) moderadamente alterado ou (3) gravemente alterado. Posteriormente, o diagnóstico final foi indicado como (0) normal, (1) disartria leve, (2) disartria moderada ou (3) disartria grave.

#### Análise acústica

A análise acústica da fala foi realizada no Praat^(40)^ (versão 6.1.55) com *script* ^(41)^ para detecção automática dos picos de intensidade. Como o português brasileiro só permite vogais no núcleo da sílaba, contar os picos de intensidade equivale a determinar o número de sílabas. A confiabilidade desse roteiro foi avaliada por De Jong e Wempe^(41)^ comparando os resultados das análises feitas e realizadas manualmente com o *script* do PRAAT utilizado neste estudo. Os parâmetros analisados foram baseados em Rusz et al.^(42)^ e Vogel e Maruff^(43)^. Para a fonação, as variáveis foram Jitter (rap), Shimmer (local), Frequência Fundamental (F_0_ em Hz), desvio padrão de F_0_ e relação harmônico-ruído (HNR), medida em vogal sustentada [a]. Para caracterizar a articulação, foram utilizadas as seguintes variáveis, obtidas nas tarefas de diadococinesia (DDK) e fala espontânea: número de sílabas, número de pausas, duração total (em segundos), *phonation time* (duração total menos pausas), *phonation rate* (*phonation time* dividido pela duração total), *speech rate* (número de sílabas dividido pela duração total), *articulation rate* (número de sílabas dividido pelo *phonation time*), duração média silábica (ASD) e número de pausas ponderadas pelo tempo total. A razão entre o 2º formante da vogal [i] e o 2º formante da vogal [u] é uma medida de centralização vocálica, que indica uma amplitude reduzida de movimentos articulatórios e é obtida a partir de uma sequência de [iu] repetidos. O tempo máximo de fonação (TMF) foi a tarefa utilizada para análise do subsistema respiratório na fala, pois esta tarefa permite analisar a relação aerodinâmica-mioelástica para fonação, embora não seja uma medida respiratória isolada, uma vez que inclui produção vocal. Quanto à prosódia, a variação de F_0_ e a variação de intensidade foram avaliadas durante a produção de uma frase com entonação afirmativa, interrogativa e exclamativa. A variação de F_0_, ou seja, a diferença entre os valores máximo e mínimo de F_0_, indica a variação melódica e, portanto, a capacidade do locutor de variar a entonação (Figura 1).

**Figura 1:** Desenho Experimental

### Análise estatística

Realizou-se uma análise qualitativa dos dados utilizando intervalos interquartis e mediana. Os dados foram analisados quantitativamente pelo coeficiente de correlação de postos de Spearman. Para a análise de comparação entre os grupos foram feitas as médias, os desvios padrões e aplicado o teste t.

## RESULTADOS

Foram incluídos no estudo sete indivíduos, sendo 4 mulheres e 3 homens, pareados por idade e sexo com controles. Na Tabela 1, descrevemos as características clínicas e sociodemográficas da amostra sem diferenças estatisticamente significativas entre os grupos.

**Tabela 1:** Variáveis demográficas e clínicas

Variável	Q1	Mediana	Q3
Idade	40	48	64
Escolaridade	7	9	11
CAGexp	43	47	47
Idade de início	30	45	50
Duração da doença	3	6	9

**Legenda:** Q1 = primeiro intervalo interquartil; Q3 = terceiro intervalo interquartil; CAGexp = repetições do alelo CAG expandido

Na Tabela 2, analisamos as escalas de gravidade quanto a questões funcionais, comportamentais e de independência (Unified Huntington's Disease Rating Scale) e as escalas cognitivas.

**Tabela 2:** Escalas de gravidade e cognitivas

Variável	Q1	Mediana	Q3
UHDRS motor	30	41	68
UHDRS comportamental	4	11	37
Escala capacidade	8	8	18
Escala de independência	60	70	70
Escala funcional	3	4	5
Fluência verbal fonológica	9,5	11	13
Fluência verbal categórica	6	8	9,5
MoCA	10	12,5	18,5
Símbolos acertos	0,75	8,5	24,25
FAB	4,75	8,5	10,5
Stroop leitura	26	38,5	90,75
Stroop nomeação	27,5	46	88
Stroop interferência	11	12,5	32,5

**Legenda:** Q1 = primeiro intervalo interquartil; Q3 = terceiro intervalo interquartil; UHDRS = Unified Huntington's Disease Rating Scale; MoCA = Avaliação Cognitiva de Montreal; FAB = Bateria de Avaliação Frontal

No Quadro 1 foi descrito o julgamento perceptivo auditivo da fala. Todos os indivíduos apresentaram restrição prosódica e imprecisão articulatória, e 6 indivíduos apresentaram incoordenação pneumofonoarticulatória e lentidão.

**Quadro 1:** Características de fala pelo julgamento perceptivo-auditivo

**1**	**Sexo**	**Idade (anos)**	**Educação (anos)**	**Tempo de doença (anos)**	**Idade de início dos sintomas (anos)**	**Grau de disartria**	**Características de fala**	**% Inteligibilidade**
**HD1**	**Masculino**	**64**	**9**	**14**	**50**	**Grave**	*Monopitch*, *monoloudness*, restrição prosódica, hipofonia, incoordenação pneumofonoarticulatória, imprecisão articulatória, lentidão	**50%**
**HD2**	**Feminino**	**40**	**11**	**10**	**30**	**Moderado**	restrição prosódica, incoordenação pneumofonoarticulatória, imprecisão articulatória, lentidão	**60%**
**HD3**	**Feminino**	**48**	**11**	**3**	**45**	**Leve**	restrição prosódica, imprecisão articulatória	**75%**
**HD4**	**Feminino**	**54**	**8**	**4**	**50**	**Grave**	*monopitch*, *monoloudness*, restrição prosódica, hipofonia, incoordenação pneumofonoarticulatória, imprecisão articulatória, lentidão, prolongamento de fonemas, disfluência	**50%**
**HD5**	**Masculino**	**23**	**7**	**7**	**16**	**Grave**	*monopitch*, *monoloudness*, restrição prosódica, hipofonia, incoordenação pneumofonoarticulatória, imprecisão articulatória, lentidão	**45%**
**HD6**	**Masculino**	**42**	**11**	**6**	**36**	**Moderado**	Alteração vocal, restrição prosódica, hipofonia, incoordenação pneumofonoarticulatória, imprecisão articulatória, lentidão	**50%**
**HD7**	**Feminino**	**71**	**5**	**5**	**66**	**Grave**	restrição prosódica, incoordenação pneumofonoarticulatória, imprecisão articulatória, lentidão, prolongamento de fonemas	**45%**

Fonte: Desenvolvido pelo autor

Na Tabela 3 apresentamos a comparação das variáveis da análise acústica da fala entre casos e controles nos subsistemas: fonação, respiração e prosódia. Os indivíduos com DH foram significativamente piores do que o grupo controle nas variáveis de frequência fundamental, tempo máximo fonatório, *jitter* local, *shimmer* local, variação de frequência fundamental na frase afirmativa, e variação de intensidade nas frases interrogativa e exclamativa.

**Tabela 3:** Comparação das variáveis da análise acústica da fala entre caso e controle nas bases motoras da fonação, respiração e prosódia

**Base Motora**	**Variáveis**	**Caso**	**Controle**	**p-valor**
Fonação	*Jitter local*	0,87(±0,49)	0,39(±0,10)	0,04
	*Shimmer local*	8,47(±4,92)	3,59(±2,72)	0,046
	FF média	215,99(±70,15)	154,53(±43,21)	ns
	FF mínimo	174,45(±63,13)	149,62(±43,04)	ns
	FF máximo	297,78(±108,60)	160,42(±44,13)	0,015
	FF desvio padrão	21,88(±21,24)	1,62(±0,50)	0,045
	HNR	17,32(±7,16)	20,75(±4,23)	ns
Respiração	TMF	5,45(±5,03)	17,22(±4,43)	0,001
Prosódia	Variação de F0 afirmação	221,26(±112,95)	461,62(±176,52)	0,012
	Variação de intensidade afirmação	48,58(±20,37)	30,43(±6,52)	ns
	Variação de F0 interrogativa	194,36(±111,61)	361,67(±197,63)	ns
	Variação de intensidade interrogativa	47,68(±18,73)	29,39(±5,92)	0,042
	Variação de F0 exclamativa	206,93(±132,22)	390,41(±184,24)	ns
	Variação de intensidade exclamativa	47,72(±13,78)	33,03(±9,65)	0,042

**Legenda:** F2 = frequência do segundo formante; F2i/F2u = frequência do Segundo formante no [i] dividida pela frequência do segundo formante [u] em segundos; FF = frequência fundamental; TMF = tempo máximo de fonação; HNR = relação harmônico-ruído; ASD = duração média das sílabas na fala espontânea; Ns = não significativo estatisticamente

Na Tabela 4 apresentamos a comparação das variáveis da análise acústica da fala entre casos e controles no subsistema articulação. Os indivíduos com DH foram significativamente piores do que o grupo controle nas variáveis *speech rate*, tempo de fonação na fala espontânea, número de sílabas, média de duração das sílabas e na duração da fala espontânea.

**Tabela 4:** Comparação das variáveis da análise acústica da fala entre caso e controle na base motora da articulação

	Variáveis	Caso	Controle	p-valor
Ditongo /iu/ repetidamente	F2I/F2U	2,37(±0,48)	2,52(±0,30)	ns
Diadococinesia /pa-ta-ka/	Número de sílabas	22,86(±24,15)	48,57(±13,60)	0,035
	Número de pausas	2,00(±2,08)	0,29(±0,76)	Ns
	Duração	6,38(±4,63)	9,65(±2,72)	Ns
	*Phonation time*	5,05(±4,63)	9,55(±2,58)	0,05
	*Speech rate*	3,39(±1,15)	5,28(±1,60)	0,028
	*Articulation rate*	4,34(±0,53)	5,30(±1,56)	Ns
	ASD	0,23(±0,03)	0,21(±0,07)	Ns
Fala espontânea	Número de sílabas	76,86(±25,58)	195,29(±36,82)	<0,0001
	Número de pausas	10,43(±2,23)	12,71(±6,60)	ns
	Duração	30,86(±1,77)	59,78(±0,59)	<0,0001
	*Phonation time*	17,72(±6,11)	49,98(±5,86)	<0,0001
	*Speech rate*	2,49(±0,83)	3,27(±0,62)	ns
	*Articulation rate*	4,44(±0,88)	3,89(±0,48)	ns
	ASD	0,23(±0,05)	0,26(±0,03)	ns

**Legenda:** ASD = duração média das sílabas na fala espontânea; Ns = não significativo estatisticamente

Na Tabela 5, descrevemos as correlações que apresentaram algum grau de significância, entre a fala e os aspectos clínicos.

**Tabela 5:** Dados correlacionados teste de Spearman

Variáveis	Escolaridade (anos)	CAGexp	Duração (anos)	UHDRS motor
	Correlação de Spearman rho (p)			
Grau da disartria no JPA	-0,868 (0,011)	NS	NS	NS
F2i	NS	NS	NS	-0,821 (0,023)
FF máximo	NS	NS	NS	-0,929 (0,003)
HNR	NS	0,852 (0,015)	NS	NS
Diadococinesia				
*Número de sílabas*	NS	NS	NS	-0,800 (0,031)
*Speech rate*	NS	NS	NS	-0,857 (0,014)
Fala espontânea				
*Número de pausas*	NS	NS	NS	NS
Intensidade mínima frase interrogativa	0,778 (0,039)	NS	NS	NS
Intensidade mínima frase exclamativa	NS	NS	NS	-0,893 (0,007)
Variação de intensidade frase exclamativa	NS	NS	0,775 (0,041)	0,929 (0,003)
TMF	NS	NS	NS	-0,893 (0,007)

**Legenda:** JPA = julgamento perceptivo-auditivo; F2 [i] = frequência do segundo formante no [i]; FF = frequência fundamental máxima; HNR = relação harmônico-ruído; TMF = tempo máximo de fonação; NS = não significativo estatisticamente

## DISCUSSÃO

Neste estudo, todos os indivíduos com DH foram diagnosticados com algum grau de disartria, sendo 4 (57,15%) de grau grave, 2 (28,57%) de grau moderado e 1 (14,28%) de grau leve. No julgamento perceptivo auditivo da fala, dentre os parâmetros avaliados, as alterações mais frequentes foram restrição prosódica, imprecisão articulatória, incoordenação pneumofonoarticulatória e lentidão.

Em relação às variáveis acústicas da fala, os indivíduos com DH foram significativamente piores do que o grupo controle na fonação (frequência fundamental, tempo de fonação, *jitter* local, *shimmer* local), respiração (tempo máximo de fonação) e articulação (*speech rate*, tempo de fonação na fala espontânea, número de sílabas na fala espontânea, média de duração das sílabas e na duração da fala espontânea).

A literatura disponível^(21,23,24,28)^ que aborda sobre a fala na doença de Huntington descreve a linguagem, tendo como exemplo a análise do discurso, e a fala a partir de análise das bases motoras. Observou-se que todos os artigos analisam a fala dos indivíduos nos graus leves da doença e utilizam diferentes softwares para análise perceptivo-auditivas ou análises acústicas. Os estudos descrevem que o perfil de fala dessa população é caracterizado por tempo de início de fala aumentado, prolongamento de vogais, menor taxa de articulação e de velocidade de fala, presença de interrupções súbitas durante a fonação e imprecisões articulatórias.

Hertrich e Ackermann^(23)^ realizaram avaliação acústica em 13 indivíduos com DH (a maioria em fases mais avançadas) e 12 controles. Os indivíduos com DH mostraram aumento da variabilidade acústica e do tempo para início da fala (voice-onset-time), além de prolongamento excessivo de vogais curtas. Os autores interpretaram esses achados como decorrentes da doença avançada, o que foi congruente com a literatura para patologias degenerativas cerebelares. Em nosso estudo, nos indivíduos com DH observamos um número maior de pausas durante a fala, o tempo de fonação menor bem como a diminuição do número de sílabas por segundo mostrando-nos um perfil semelhante ao estudo mencionado anteriormente mesmo que com um número menor de indivíduos analisados.

Outro estudo avaliou 21 indivíduos com DH (5 anos de duração em média) e 21 controles. Os autores descrevem um padrão de alteração com redução da velocidade da fala (taxa de articulação), aumento das pausas e grande incapacidade na geração de sílabas únicas^(24)^. O presente estudo também identificou nos indivíduos com DH a redução na velocidade de fala baseada na taxa de articulação, além disso identificou que o número de pausas na articulação foi expressivamente maior quando comparado a outros estudos semelhantes. Nossas hipóteses para esses achados incluem a baixa escolaridade da população estudada, assim como alterações cognitivas e linguísticas associadas. Além disso, a idade, a idade de início e o tempo de duração da doença podem exercer influência sobre essas variáveis.

Foram encontradas correlações entre os aspectos clínicos dos sujeitos com DH e o perfil de fala, tais como a relação entre os escores da escala motora e as bases motoras da fala. Dessa forma, verificamos nessa amostra que, quanto pior o sujeito está motoramente, maior o prejuízo em todas as bases motoras da fala. Não houve correlação com o perfil de fala entre as variáveis cognitivas e as outras variáveis clínicas (tempo de doença, idade de início, escala comportamental, funcional e de independência).

Rusz et al.^(25)^, avaliaram 34 indivíduos DH (com duração média da doença de 5,9 anos) e 34 controles. Os autores encontraram flutuação irregular nos tons, interrupção súbita na fonação e imprecisões articulatórias. Nesse estudo, os autores observaram moderada correlação (r = -0,48) entre interrupção súbita da fonação e componentes voluntários da escala UHDRS. Em nosso estudo, encontramos um prejuízo expressivo na taxa de sílabas por segundo, implicando imprecisões articulatórias e variações em jitter e shimmer, comprometendo a fonação.

O estudo de Illes^(44)^ analisou a fala espontânea em subgrupos de 10 sujeitos com Doença de Huntington, 10 com Doença de Alzheimer (DA) e 10 com Doença de Parkinson (DP). Relata que pacientes com DH apresentam parafasias na fala espontânea bem como redução no uso de frases complexas no momento da fala. Os resultados de Illes colaboram com os achados em nosso estudo visto o tempo de fala espontânea do grupo caso ser expressivamente menor quando comparado ao grupo controle.

Assim como os déficits de linguagem, fluência lexical reduzida e dificuldades de comunicação bastante descrita na literatura, o comprometimento cognitivo na doença de Huntington (HD) também já foi bastante caracterizada em diferentes estágios da doença, com alterações na memória, funções executivas e atenção que podem ser percebidas previamente ao diagnóstico motor^(45,46)^. As alterações encontradas na nossa amostra estão de acordo com os achados da literatura. Nossos casos tiveram no teste de rastreio MOCA^(47)^ a mediana de 12,5 pontos, estando abaixo do ponto de corte para distinguir controles saudáveis de pacientes com demência (15 pontos) (sensibilidade 90%, especificidade 77%).O mesmo ocorreu com a bateria FAB^(48)^, que avalia funções executivas, uma vez que nossos casos apresentaram uma mediana de 8,5 (conforme Tabela 2) e o ponto de corte é 13,0 (±2,3). As alterações na fluência verbal (tanto semântica como fonológica) encontrada nos pacientes podem também ter impactado na velocidade de fala.

A tarefa de DDK foi citada como importante na identificação de casos neurológicos^(49)^, uma vez que os indivíduos apresentam menos sílabas por respiração^(30)^. A fonação, no desempenho motor oral, incluindo DDK oral (articulação) e na prosódia foram as alterações mais comuns em DH^(50)^. Contudo, também identificou que as alterações de fala dos indivíduos com DH está diretamente relacionado com a evolução dos sintomas motores, sendo assim, um sinal de alerta para os profissionais encaminharem para avaliação fonoaudiológica visando a intervenção precoce visto que quanto pior os sintomas motores dos pacientes, maior será o prejuízo em todas as bases motoras da fala.

Em todas as variáveis de fala, os participantes com DH foram piores que os controles. A justificativa da inclusão de controles neste estudo foi fornecer uma referência de comparação, especialmente porque nem todas as variáveis de fala analisadas possuem um ponto de corte ou um padrão de normalidade estabelecido para a população brasileira. Além disso, a amostra sendo uma série de casos a comparação visou assegurar que as diferenças observadas foram em função da condição estudada, e não a outras variáveis externas, como, por exemplo, a escolaridade.

Uma limitação do estudo foi o número pequeno da amostra, uma vez que a DH tem baixa prevalência e foram incluídos indivíduos de somente um centro especializado. Novos estudos com amostras maiores envolvendo mais centros de cuidado e acompanhamento longitudinal de pacientes com DH seriam importantes para estabelecer um perfil de fala desses indivíduos, contribuindo com novas abordagens diagnósticas e terapêuticas.

Assim sendo, o presente estudo nos mostra que indivíduos com DH apresentam prejuízos significativos na articulação, respiração e fonação. Sendo a articulação a base motora com maior prejuízo. Considerando as alterações mencionadas anteriormente, faz-se necessário que o profissional clínico se mantenha atento a estas três bases motoras de fala no momento de avaliação e aplicação de protocolos, visto que poderão apresentar alterações significativas de resultados que definirá o percurso de reabilitação do indivíduo na clínica fonoaudiológica.

## CONCLUSÃO

Esta pesquisa identificou que as bases motoras mais afetadas em indivíduos com Doença de Huntington (DH) são articulação, fonação e respiração. Portanto, as tarefas mais indicadas para a avaliação desses indivíduos devem incluir as análises desses subsistemas da fala, sendo essas as áreas de foco prioritárias para a terapia. Além disso, foi observado que o perfil de fala está diretamente relacionado à progressão dos sintomas motores nesses sujeitos. A elaboração de um perfil de fala desses indivíduos pode proporcionar novas abordagens no diagnóstico e reabilitação precoce, contribuindo para uma melhor qualidade de vida. A generalização dos dados apresentados neste estudo não é possível devido ao tamanho da amostra, porém, a avaliação da fala mostra-se como possibilidade de um potencial biomarcador que pode se configurar como um dos preditores da progressão da doença.
